# Photon-counting detector CT in oncology: a new era of cancer imaging

**DOI:** 10.1186/s13244-025-02176-2

**Published:** 2026-01-21

**Authors:** Elisa Bruno, Anna Palmisano, Enrico Camisassa, Davide Vignale, Carlo Tacchetti, Antonio Esposito

**Affiliations:** 1 Advanced Imaging for Personalized Medicine Unit, Experimental Imaging Center, IRCCS San Raffaele Hospital, Milan, Italy; 2 Vita-Salute San Raffaele University, Milan, Italy

**Keywords:** Photon-counting detector CT, Cancer, Spectral, Energy integrated detector CT, Oncology

## Abstract

**Abstract:**

Oncologic imaging plays a critical role in the diagnosis, staging, treatment planning, and follow-up of cancer patients. Recent advancements in computed tomography, particularly the development of photon-counting detector CT (PCCT), have introduced new opportunities for improving diagnostic accuracy and tissue characterization, while reducing contrast agent usage and radiation exposure. By offering ultra-high spatial resolution, enhanced contrast-to-noise ratio, and intrinsic spectral capabilities, PCCT addresses many limitations of conventional energy-integrating detector CT (EID-CT) and unlocks new possibilities for quantitative imaging. This review explores the emerging applications of PCCT across various tumor types—including thoracic, abdominal, and musculoskeletal malignancies—highlighting its potential to improve cancer imaging and patient care.

**Critical relevance statement:**

Photon-counting detector CT (PCCT) offers several advantages in oncologic imaging, providing superior spatial resolution, spectral imaging capabilities, and reduced radiation dose, enhancing lesion characterization and precise treatment planning, making PCCT a valuable tool for personalized cancer care.

**Key Points:**

- CT has a crucial role in oncological imaging, supporting diagnosis, staging, treatment planning and follow-up.
- Compared to EID-CT, PCCT offers higher spatial and contrast resolution, reduces artifacts and image noise and provides spectral data enabling quantitative assessment.
- PCCT may improve cancer imaging by increasing diagnostic accuracy, with better detection of small lesions, enhanced soft tissue contrast, and enabling quantitative iodine uptake evaluation.

**Graphical Abstract:**

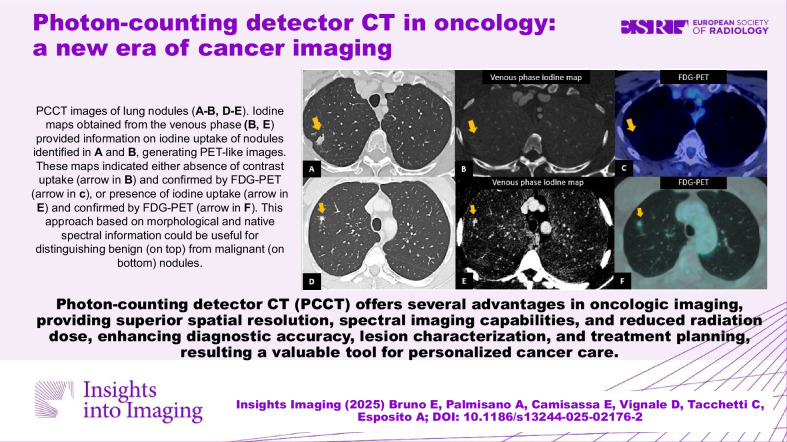

## Introduction

Cancer is a growing cause of mortality. According to the WHO report, 20 million new cancer cases were diagnosed in 2022, mainly involving lung, breast and colon, and 9.7 million people died from oncological diseases. Moreover, an estimated 53.5 million individuals were alive within 5 years of a cancer diagnosis [1]. Therefore, early diagnosis, accurate staging and monitoring are crucial for effective and personalized treatment.

CT plays a pivotal role in oncology by providing detailed cross-sectional images essential for diagnosis, staging, treatment planning, and follow-up [2].

Since its introduction into clinical practice in the early 1970s, CT technology has rapidly evolved, with significant improvements in spatial and temporal resolution. The advent of multidetector CT marked a major milestone, enabling wide anatomical coverage in a short scanning time and delivering high-quality images for cancer management [3, 4]. Later, the introduction of dual-energy CT (DECT) opened new possibilities for quantitative tissue characterization by measuring iodine uptake, as well as for enhancing image quality through virtual monoenergetic imaging (VMI) [2, 5].

This advancement paved the way for a shift in the CT’s role in oncologic imaging, from a purely anatomical tool for lesion detection and measurement, to a modality capable of evaluating tumor-associated physiological processes [6]. However, the broader application of DECT has been limited by different factors, with the main constraint being the associated increase in radiation exposure, as well as other technical and protocol-related compromises.

This paradigm shift became reality after the introduction of the photon-counting detector CT (PCCT), which stands as one of the most significant innovations in CT imaging to date [7].

PCCT counts photons individually and sorts them into distinct energy bins at the detector level, providing native spectral information, simultaneous multi-energy acquisition and k-edge imaging. Additionally, PCCT offers high energetic efficiency, with reduced electronic noise and radiation exposure, and increased spatial resolution (0.16–0.2 mm^2^ and up to 40 lp/cm), without compromises in temporal resolution [8].

These technological advancements make PCCT a promising technique for oncologic imaging, overcoming the limitation of energy-integrating detector (EID) CT and potentially improving diagnostic accuracy. However, data about its application in oncology remain limited. The aim of this narrative review is to explore the clinical benefits of PCCT across various oncologic scenarios.

### Technical background

Conventional EID-CT works by converting incident X-ray photons into visible light using a scintillator. This light is then absorbed by a photodiode and transformed into a digital signal. However, since photons of varying energies are integrated together, valuable spectral information is lost, and the contribution of low-energy photons is underestimated, leading to reduced image contrast and increased electronic noise. Additionally, EID-CT systems require light-blocking septa between scintillator elements to prevent optical crosstalk, resulting in “dead areas” that reduce both spatial resolution and dose efficiency [9].

PCCT utilizes semiconductor detectors that directly convert individual X-ray photons into electrical pulses. These pulses are proportional to the energy of each photon and can be sorted into discrete energy bins, enabling enhanced spectral separation and more accurate tissue characterization.

Key advantages of PCCT include the elimination of septa, which allows for higher spatial resolution, the absence of the “energy weighting effect,” improving sensitivity to low-energy photons. Furthermore, the ability to suppress subthreshold electrical signals effectively eliminates electronic noise, thereby enhancing soft tissue contrast and dose efficiency [10].

### Spatial resolution

PCCT’s improved spatial resolution arises from overcoming limitations of EID-CT detectors, including the minimal size of detector elements and the requirement for septa. PCCT achieves up to an isotropic resolution of 0.2 mm and a 1024 × 1024 matrix.

The FDA-approved pixel sizes at the isocenter of 0.151 × 0.176 mm² (sub-pixel) and 0.302 × 0.352 mm² (macro-pixel), enabling spatial resolution up to 40 lp/cm, compared to 20 lp/cm for EID-CT (0.625 × 0.625 mm²) [11]. Additionally, the direct conversion of X-ray photons into an electrical signal eliminates the need for reflective septa. In EID-CT, the signal is generated by summing the energies of incoming photons, without differentiating their energy levels, whereas in PCCT, all photons are individually classified according to their energy.

In PCCT, low-energy photons, being closer to iodine’s k-edge (33 keV), enhance contrast through the photoelectric effect, resulting in improved contrast resolution and enhanced soft tissue visualization at equivalent iodine concentration [9, 12].

In fact, Hagen et al demonstrated a 27% reduction in contrast media and up to 34% radiation dose in obese patients, while maintaining a stable contrast-to-noise ratio [13].

### Multi-energy imaging

A key innovation offered by PCCT is its energy-resolving detector technology, which provides intrinsic spectral data that enables both VMI and material decomposition. Low-keV VMI offers a higher contrast-to-noise ratio compared to EID-CT, resulting in enhanced image quality and improved lesion detection [14].

Material decomposition allows the generation of virtual non-contrast images, virtual non-calcium images, as well as quantification maps for materials such as iodine, uric acid and calcium [11, 15].

PCCT also supports K-edge imaging, enabling the differentiation of materials like bismuth, gold, and platinum. This opens new possibilities in molecular imaging with targeted contrast agents, such as nanoparticle-based agents specifically designed for molecular targeting [8].

### Radiation dose reduction

The absence of detector septa, reduction of electrical noise, and the absence of energy weighting effect make PCCT more dose efficient with a dose reduction of approximately 32% in contrast-enhanced abdominal CT [16], and up to 66% in chest CT [17, 18].

### Noise reduction

#### PCCT reduces image noise and enhances overall image quality primarily by rejecting electronic noise

**Fig. 1:**
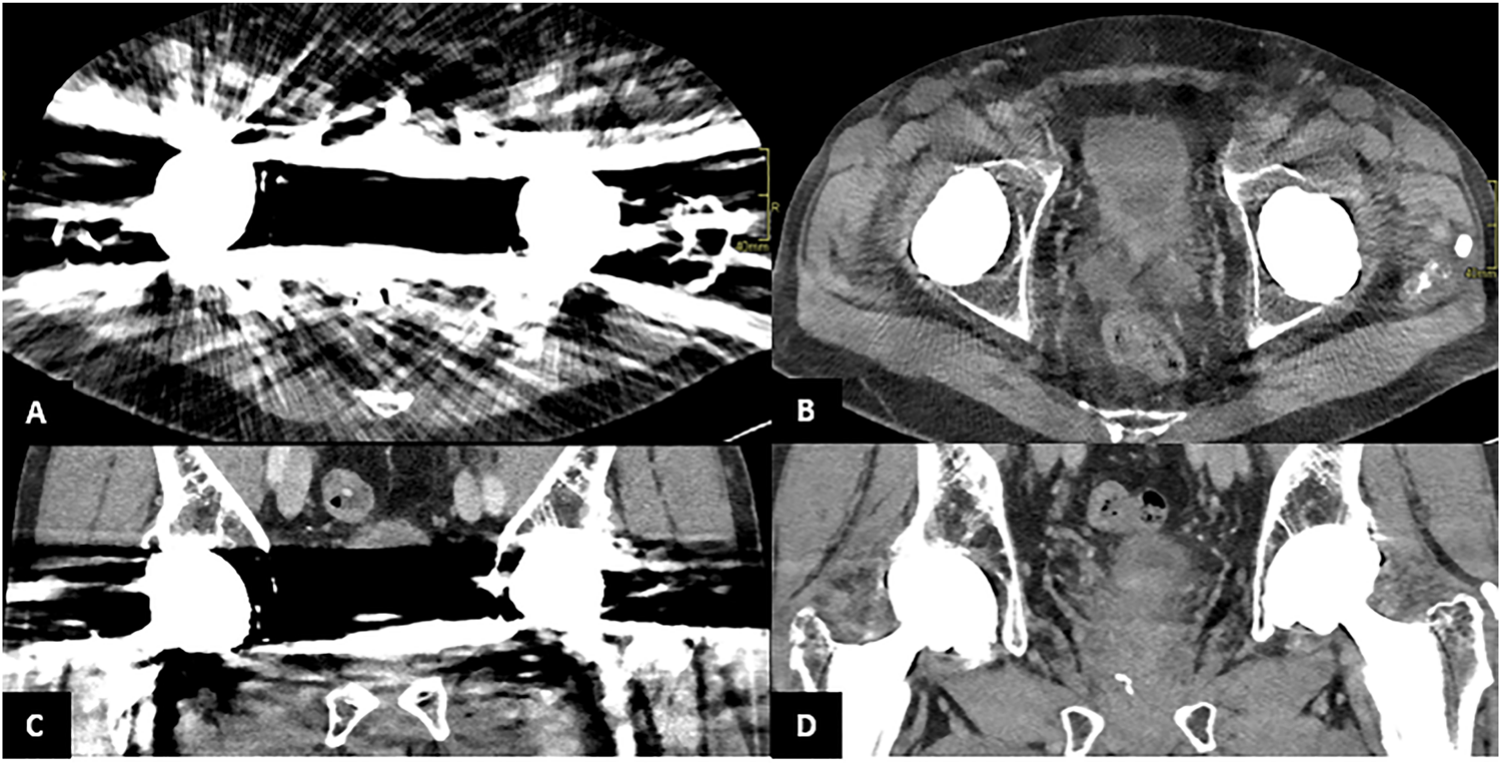
Patient with bilateral hip prosthesis undergoing PCCT scan. Axial and coronal views (**A**, **C**) show severe metal artifacts. Axial and coronal reconstructions after energy filtering (**B**, **D**) demonstrate a significant reduction in metal artifacts

It employs a thresholding method that filters out signals below 20 keV, effectively eliminating electronic noise, which typically falls below this threshold [15]. In contrast, EID-CT is affected by noise from beam hardening, caused by the attenuation of low-energy photons in dense tissues, and metal artifacts. PCCT addresses these issues by utilizing a combination of high-energy imaging and advanced filtering techniques [11] (Fig. 1).

## Clinical applications

### Lung

PCCT is particularly suitable for lung imaging due to its ability to provide ultra-high resolution images based on a higher matrix size (e.g., 1024–2048) and reduced section thickness (e.g., as low as approximately 0.2 mm), with a lower or similar radiation dose compared to EID-CT [19]. This results in better depiction of all the anatomical components in the lungs [20] and of lung disease, including emphysema, interstitial alteration including reticulation, ground glass opacity, mosaic pattern [21] nodules and masses [22]. In particular, in the setting of lung nodules, a recent meta-analysis [23] documented increased accuracy in detection, mainly related to improved image quality. In fact, PCCT was found to provide better image quality in 30.5% [24] to 54% of lung nodules [23], allowing for a better morphological evaluation [25], with more accurate identification of irregular or spiculated margins, and of distortion of adjacent vessels [25–27].

In a phantom study, Si-Mohamed et al [28] demonstrated a five times higher detectability index for a ground-glass nodule of 4 mm without an increase in radiation dose compared with DECT. Moreover, PCCT was found to improve characterization of the inner composition of lung nodules, depicting densitometric heterogeneity that is particularly useful in the characterization of subsolid nodules, as demonstrated by Wang et al [29], where the depiction of the heterogeneity of the inner solid component had prognostic implications.

Furthermore, the possibility to reduce for more than 30% the radiation dose, suggested PCCT suitability for cancer screening. In a recent study on 101 patients, Kerber et al [30] compared low-dose and ultra-low dose PCCT for cancer screening (0.11 ± 0.03 mGy, compared to 0.65 ± 0.15 mGy, *p* < 0.001) and found the efficacy of PCCT chest study acquired with X-ray dose scans for nodule detection. Accurate semiautomated and manual nodule measurements resulted feasible, despite a tendency to underestimate nodule density.

**Fig. 2:**
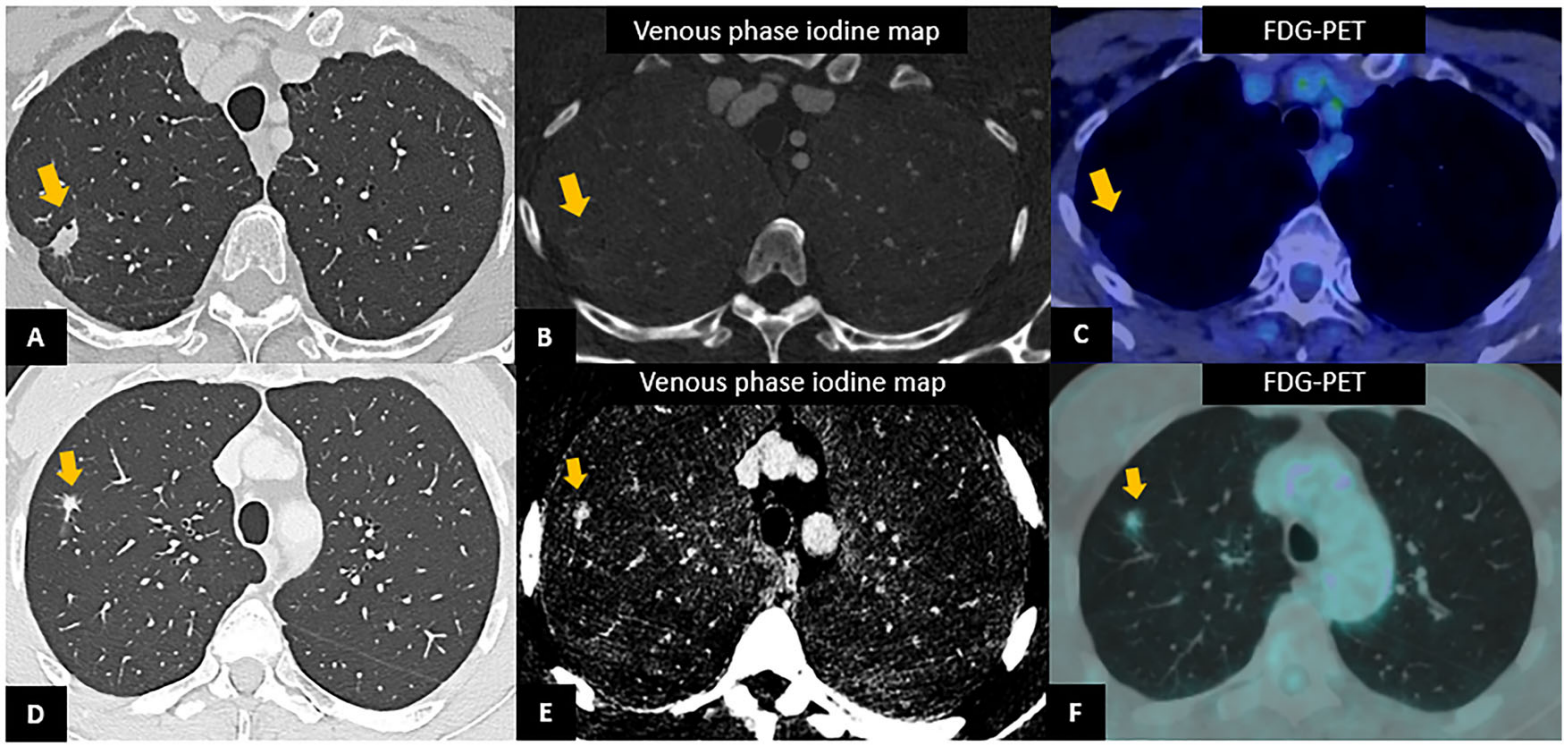
PCCT in the assessment of lung nodules. In a 62-year-old woman, chest PCCT (**A**) shows a lung parenchymal irregular nodule connected to the parietal pleura of the upper right lobe, without contrast uptake on the iodine map (**B**), resulting suggestive of a benign nodule. FDG-PET exam confirmed the benign nature for absent FDG uptake (**C**). In a 62-year-old man, PCCT shows the presence of an area of structural alteration of the lung parenchyma in the context of the right upper lung lobe, in the subpleural location, characterized by ground-glass pattern with overall size of 21 × 10 mm (**D**), containing a completely solid nodular component approximately of 1 cm in diameter, with contrast uptake on the venous iodine map, suggestive for cancer (**E**). FDG-PET confirmed the presence of an FDG avid nodule (**F**). At histological examination, it was diagnosed as lung lepidic adenocarcinoma (pT1a pN0)

In addition to increased spatial resolution, the availability of spectral information, particularly iodine maps, may improve diagnostic accuracy. Iodine maps may provide PET-like information with the possibility to discriminate benign from malignant nodules, as shown in Fig. 2. However, systematic studies in this setting still lack. To date, Huisinga et al [31] were the first to investigate the potential role of PCCT iodine map in the discrimination and characterization of different lung opacifications in clinical settings. They reported a rate of 15% of cases in which the classification of underlying pathology was changed after evaluation of iodine maps, most of which were deemed clinically significant and related to distinguishing active inflammation from fibrosis. Unfortunately, their study did not include lung cancer.

**Fig. 3:**
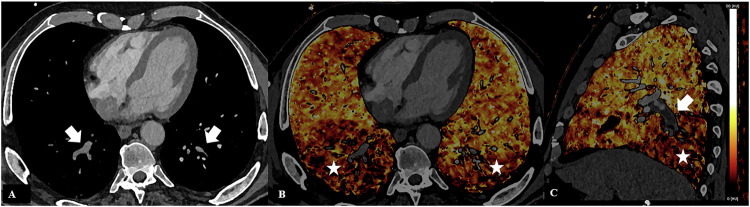
PCCT iodine perfusion maps of a patient with pulmonary artery thromboembolism. Extensive thromboembolic filling defect affecting the pulmonary artery branch for the lower right lobe (**A**) involving all segmental branches except for the upper segmental branch, with extensive hypoperfusion of the inferior right lobe (white star in **B** and **C**), indicative of ischemia. Small subsegmental thromboembolic filling defect affecting a segmental branch of the left lower lobar artery (white arrows in **A**), with associated mild peripheral hypoperfusion of the inferior left lobe (white star in **C**)

In oncological patients, iodine maps would improve the capability to detect pulmonary embolism also peripheral and of small size challenging to identify with conventional CT techniques [32, 33] thanks to the combined evaluation of angiographic scan and iodine map able to depict perfusion (Fig. 3) defect with a good accuracy compared to SPECT/CT as demonstrated in a recent study on 26 patients [34] despite a tendency to underestimated perfusion in the right middle and a tendency to overestimate it in the upper lobes.

### Breast

Among the available breast imaging modalities, MRI is the most sensitive for breast cancer detection, demonstrating superiority in primary tumor size estimation, multifocal and multicentric small disease foci, compared to mammography and US. However, MRI has notable disadvantages, such as the inability to detect small calcifications, lengthy examination time, and restricted accessibility due to claustrophobia, implanted medical devices, limited availability, and high costs [35, 36].

Dedicated breast computed tomography (BCT), which may incorporate photon-counting detectors, represents a novel approach in breast imaging, though its clinical use remains limited. By directly imaging the breast while sparing the rest of the body, BCT overcomes known drawbacks of conventional CT, including limited spatial resolution, high image noise and significant radiation exposure, which have historically hindered the use of CT in breast imaging [37].

With advancements introduced with PCCT, ultra-high-resolution breast CT imaging is now feasible, offering reduced image noise and a radiation dose comparable to or lower than that of a standard chest CT, thus enabling the visualization of breast microcalcifications [37].

In a recent study using breast phantom, Sawall et al [38] investigated the image quality of clinical PCCT compared to a dedicated breast CT system for visualizing several breast structures, such as masses and calcifications, while maintaining the same radiation dose. Despite increased noise when imaging both breast and thoracic phantoms together, PCCT demonstrated superior image quality over BCT in terms of dose-normalized contrast-to-noise ratio. Both imaging systems reliably detected calcifications as small as 0.29 mm and fibers down to 0.23 mm.

**Fig. 4:**
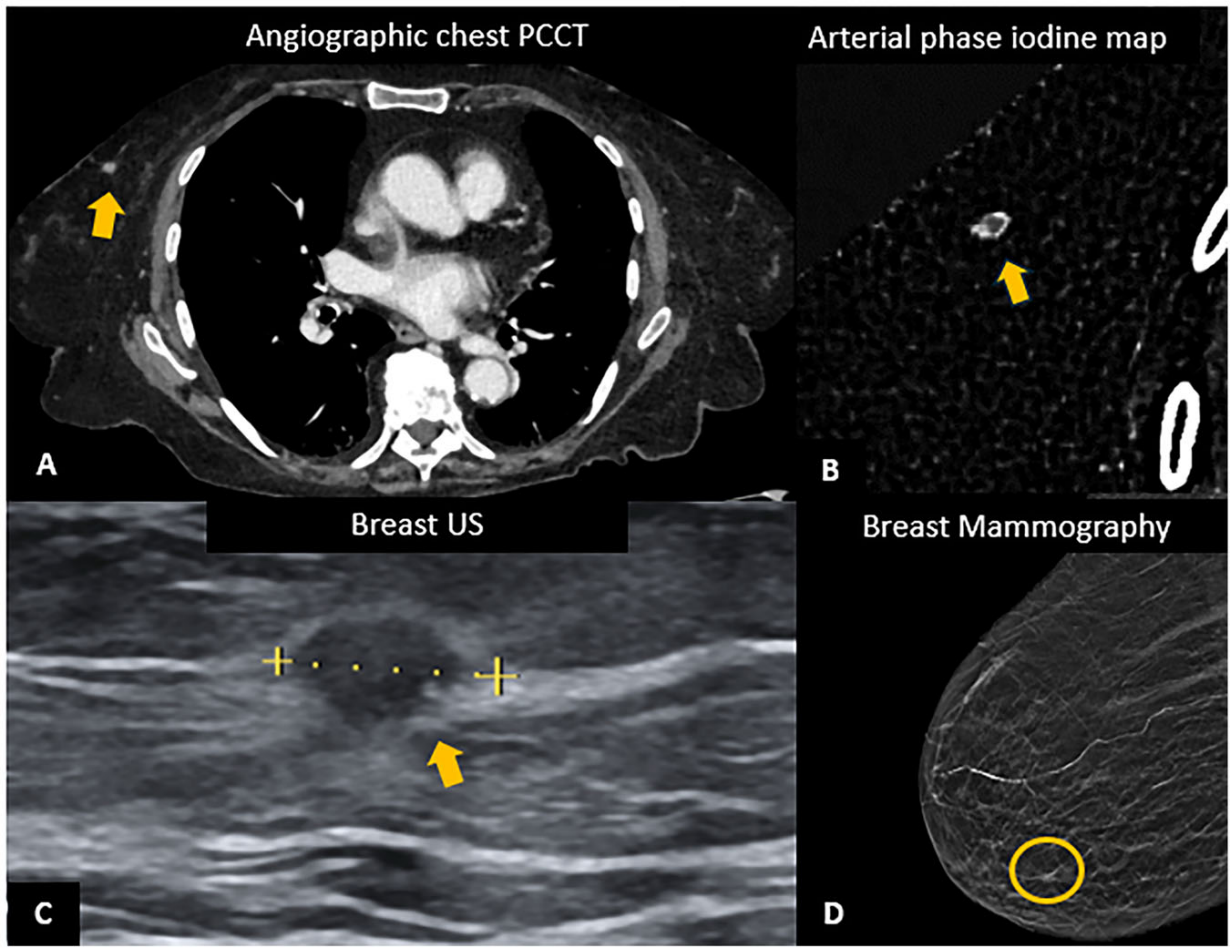
PCCT in breast cancer. Chest PCCT of a 55-year-old woman suffering from dyspnea: **A** angiographic chest examination showing an incidental finding of a mammary nodule in the lower inner quadrant of the right breast, with a diameter of 8 mm, with rim enhancement at arterial-phase iodine map (**B**). For the suspicious enhancement pattern, breast ultrasound and mammography were suggested. On the same side, breast mammography shows a small speculated margin opacity containing rare microcalcifications (**D**), and ultrasound (**C**) found a solid, hypoechoic nodule with ill-defined margins, suspected for neoplasia. Ultrasound-guided core needle biopsy was performed. At histological examination, it resulted in an invasive breast carcinoma of no special type

Beyond its high spatial resolution, PCCT also has the advantage of increased iodine attenuation, particularly using iodine maps, which are able to improve the identification of contrast-enhanced lesions (Fig. 4) with an image quality similar to the one obtained with subtraction imaging at MRI [39]. Neubauer et al [40] investigated the potential of PCCT for opportunistic locoregional breast cancer staging compared to digital mammography (DM). Their study showed significantly improved accuracy in T-classification (0.94 for PCCT vs. 0.50 for DM; *p* < 0.01), a stronger correlation in detecting the number of tumor masses (0.72 for PCCT vs. 0.50 for DM; *p* < 0.01), and better agreement with the reference standard in assessing tumor pattern and extent (0.56 for PCCT vs. 0.46 for DM; *p* < 0.01).

In a recent study on 13 patients with biopsy-proven breast cancer, Yalon et al [35] evaluated a 3-phase contrast-enhanced breast PCCT protocol against dynamic breast MRI and digital mammography. PCCT identified a suspicious mass or non-mass enhancement in 11/13 cases compared to MRI, and 3/3 cases of multifocal or multicentric disease, including a 4 mm suspicious satellite lesion.

PCCT, as recently demonstrated in a small cohort of 9 patients, seems to be accurate in staging and restaging of cancer compared to MRI with an excellent correlation for tumor size before therapy (monoenergetic reconstruction r = 0.940, *p* < 0.001), and the iodine map (r = 0.919, *p* < 0.001), and good after therapy in comparison to histology (ρ = 0.786, *p* = 0.012). This suggests the value of PCCT in local staging. With this aim, images were acquired in standard prone breast imaging position at 85 s delay from contrast administration [39].

Even though further studies are needed, PCCT has shown promising results in detecting the primary tumor and performing locoregional staging. It could be an alternative to breast MRI, particularly for patients who cannot undergo MRI due to contraindications or claustrophobia.

### Liver

Hepatocellular carcinoma (HCC) is the most common primary liver tumor [41], and its pathognomonic behavior after contrast media injection makes it especially amenable to imaging-based diagnosis. In particular, for patients with liver cirrhosis, a hyperenhancing nodule in the arterial phase followed by non-peripheral washout allows for a cross-sectional imaging-based diagnosis of HCC without the need for histological confirmation.

However, small HCC nodules smaller than 2 cm might be challenging to diagnose due to their subtle enhancement and potential anatomic variations within a cirrhotic liver [42].

Therefore, improvements in image quality have a key role in early HCC detection [43, 44].

**Fig. 5:**
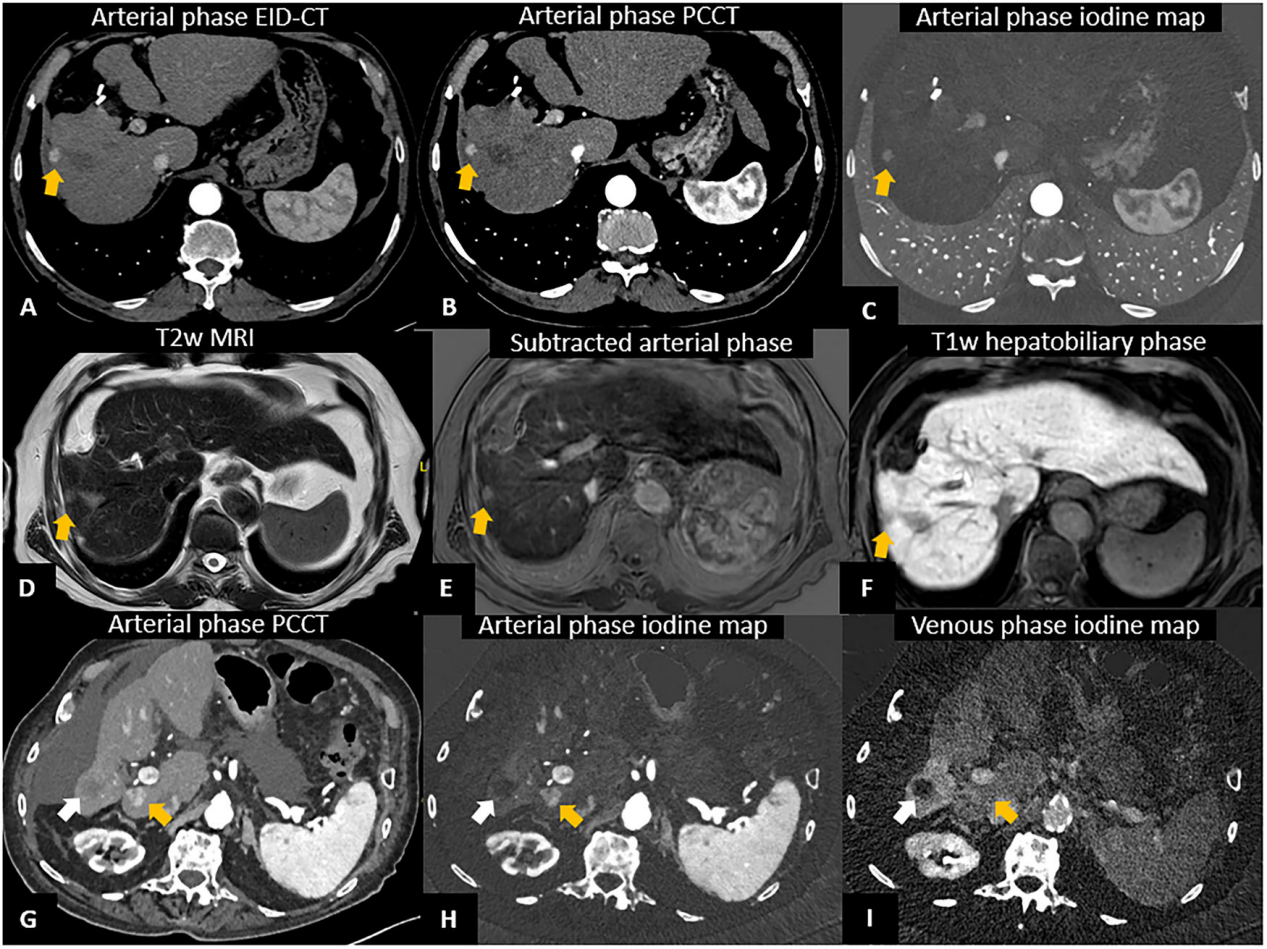
PCCT in hepatocellular carcinoma (HCC). A 75-year-old man with a previous resection for HCC and suspicion of disease recurrence. Both EID-CT (**A**) and PCCT (**B**) axial scans show a hypervascular nodule at the VI liver segment (yellow arrow in **A** and **B**), with a better margin definition at PCCT and increased iodine enhancement, further improved by iodine maps (**C**) that provided a subtraction-like image obtained from dynamic contrast enhancement MRI (**F**). PCCT findings were confirmed at MRI (**D**–**F**). A 94-year-old man with a history of HCC undergoing PCCT (**G–I**). Arterial (**H**) and venous (**I**) iodine maps show a hypodense round-shaped formation in the right hepatic lobe (white arrow in **G**–**I**), measuring 3 cm, consistent with the sequelae of HCC treated with stereotactic radiotherapy, with no enhancement suggestive for disease recurrence, but with an hypervascular nodule of VII segment (yellow arrow in **G** and **H**) with venous washout (yellow arrow in **I**) suggestive for HCC

Given the hypervascular nature of HCC, accurate assessment of the arterial phase is essential. PCCT offers the advantage of enhanced contrast resolution, particularly with low VMIs and iodine maps, which improve lesion detection and obtain MRI-like subtraction images (Fig. 5).

Low-energy VMIs have shown improved diagnostic performance in abdominal imaging, mainly due to the increased iodine attenuation near its K-edge. Graafen et al [14] reported that 50 and 60 keV VMIs from PCCT provide the best arterial-phase image quality for abdominal oncology, while Estler et al [45] found a high contrast-to-noise ratio for HCC at 40–50 keV.

In a phantom study, Racine et al [46] reported the highest detectability rate at 65 and 70 keV for both hypoattenuating and hyperattenuating lesions.

However, further studies are needed to determine the optimal keV setting for liver imaging.

Moreover, previous studies have shown that softer reconstruction kernels improve image quality and reduce noise in PCCT, particularly for evaluating HCC, facilitating planning of transarterial chemoembolization and assessing vascular invasion [47].

Liver metastases are the most common malignant liver tumors, and their early identification is crucial for patient treatment. However, conventional CT is hampered by limited spatial resolution, leading to difficulty in detecting lesions smaller than 1 cm [48].

Beyond HCC, PCCT also improves the detection of small hypovascular liver metastases compared to EID-CT [49, 50], increasing reader sensitivity and confidence in the detection of lesions, especially those smaller than 1 cm, with concurrent radiation dose reduction [51].

A recent study involving 100 patients showed a 15% higher tumor-to-liver ratio using PCCT, with the highest CNR observed below 70 keV, particularly in patients with a higher body mass index, when assessing hypovascular liver metastases [50].

These findings highlight the potential of PCCT to improve liver cancer detection, particularly in challenging cases, and suggest that further research could refine its optimal use in clinical practice.

### Pancreas

Pancreatic ductal adenocarcinoma (PDAC) is the most common primary malignant pancreatic tumor, characterized by a very low survival rate. Therefore, early diagnosis is crucial for improving prognosis [52]. It usually appears as a hypovascular mass in the pancreatic phase, and although CT is the gold standard imaging technique with a sensitivity of 76–92%, it is often difficult to detect during the early stages [53].

Currently, studies investigating the application of PCCT in pancreatic cancer imaging remain limited.

Decker et al [54] demonstrated that compared to EID-CT, PCCT VMI at 40 keV improves the conspicuity of PDAC in both the arterial and portal venous phase, potentially enhancing tumor detection in clinical practice, as later confirmed by Woeltjen et al [55].

Ruff et al identified 55 keV VMI as the optimal setting for visualizing PDAC during the pancreatic parenchymal phase with PCCT. They found that 55 keV VMI provided superior overall image quality and lower noise compared to 40 keV (mean [SE] 3.4 [0.08] vs. 2.5 [0.08], *p* < 0.001), while both energy levels yielded the highest tumor conspicuity [56].

**Fig. 6:**
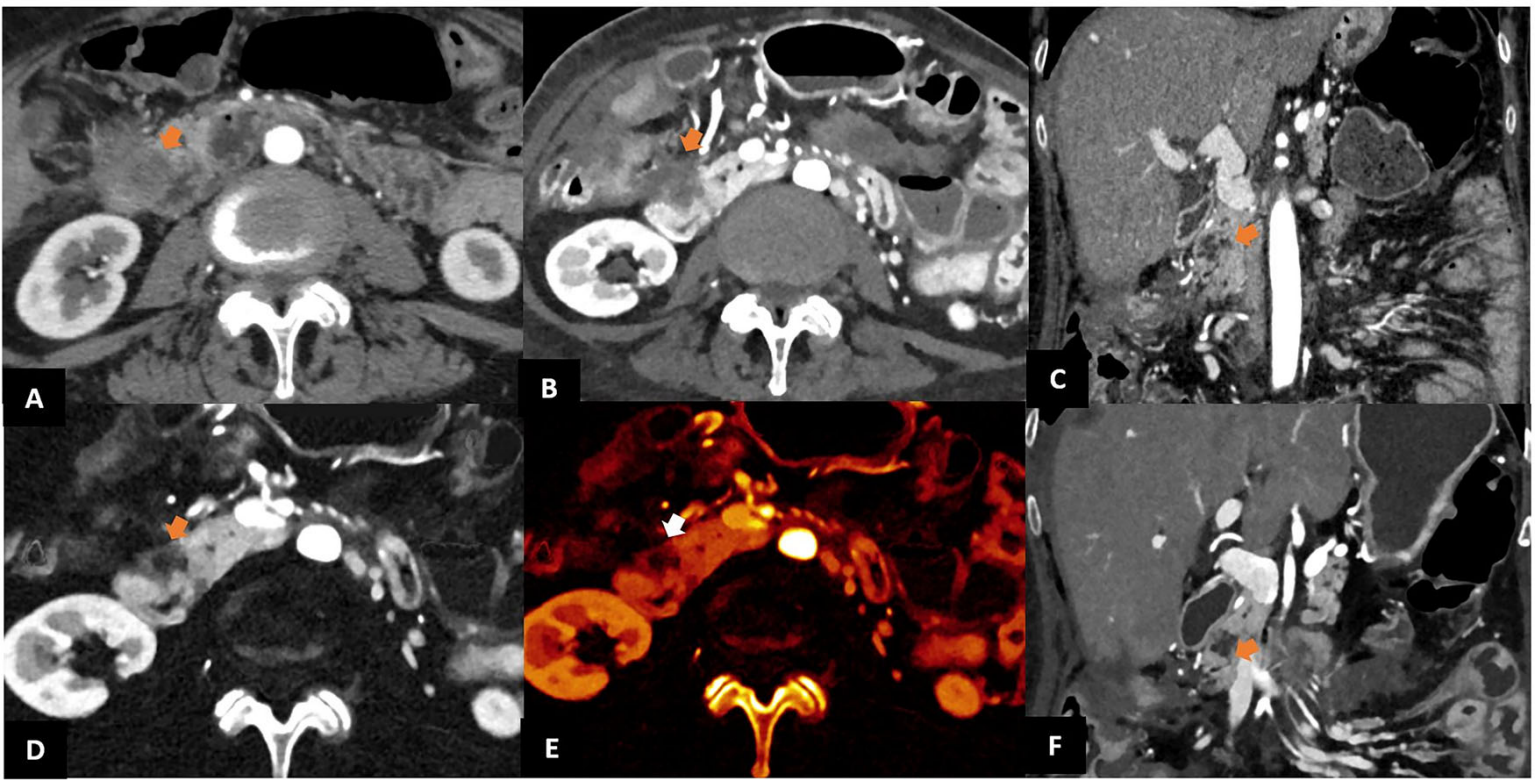
PCCT and EID-CT in pancreatic imaging. EID-CT (**A**) and PCCT (**B**) with corresponding iodine map (**D**) and color-coded iodine map (**E**) show pathological tissue measuring approximately 2.5 cm, developing from the anterior-inferior margin of the pancreatic head and infiltrating the wall of the ascending colon, the inferior duodenal flexure and the gastroduodenal artery, with better anatomical assessment on PCCT. Coronal images from EID-CT (**C**) and PCCT (**F**) demonstrate a multilocular pancreatic cystic formation. Communication with the main pancreatic duct was visible only on PCCT (arrow in **F**), allowing for a diagnosis of IPMN

Moreover, thanks to enhanced spatial and contrast resolution, PCCT may provide more detailed pancreatic imaging [57], aiding in the assessment of local invasion, perineural and vascular involvement—critical factors in determining tumor resectability [58] (Fig. 6). Indeed, a recent study comparing inter-reader agreement in assessing pancreatic tumor resectability in 145 patients found that PCCT achieved substantial inter-reader agreement for the celiac artery, superior mesenteric artery, and superior mesenteric vein (κ = 0.61–0.69), compared to moderate agreement with EID-CT (κ = 0.56–0.59) [59]. Distinguishing local tumor recurrence on surveillance CT from postoperative changes after surgical resection is also a primary challenge in patients with PDAC. Alagic et al demonstrated for the first time that spectral CT variables, when combined in a multivariable model, increase diagnostic performance, especially in the late arterial phase with 94% sensitivity, 84% specificity, and 87% accuracy [60].

PCCT’s increased spatial and contrast resolution could improve diagnostic confidence in patients with pancreatic cystic neoplasms. It has been shown to increase cyst detection compared to EID-CT [61], providing higher diagnostic accuracy (AUC: 0.81 vs. 0.74; *p* = 0.002; sensitivity: 76.8% vs. 59.4%), image quality, lesion conspicuity and diagnostic confidence at a lower radiation dose [62]. It aids in differentiating between various cystic neoplasm types and in identifying subtle enhancing mural nodules, thus providing valuable insights for clinical decision-making [63] (Fig. 6).

### Colorectal

Colorectal cancer is one of the most prevalent cancers worldwide [64]. MRI is the imaging modality of choice for its staging, as it offers high contrast resolution for soft tissues and correlates well with tumor histological grade and treatment response [65, 66].

In contrast, conventional CT has a limited role in local staging, mainly due to its lower soft tissue contrast resolution, and is primarily used to detect distant metastases.

**Fig. 7:**
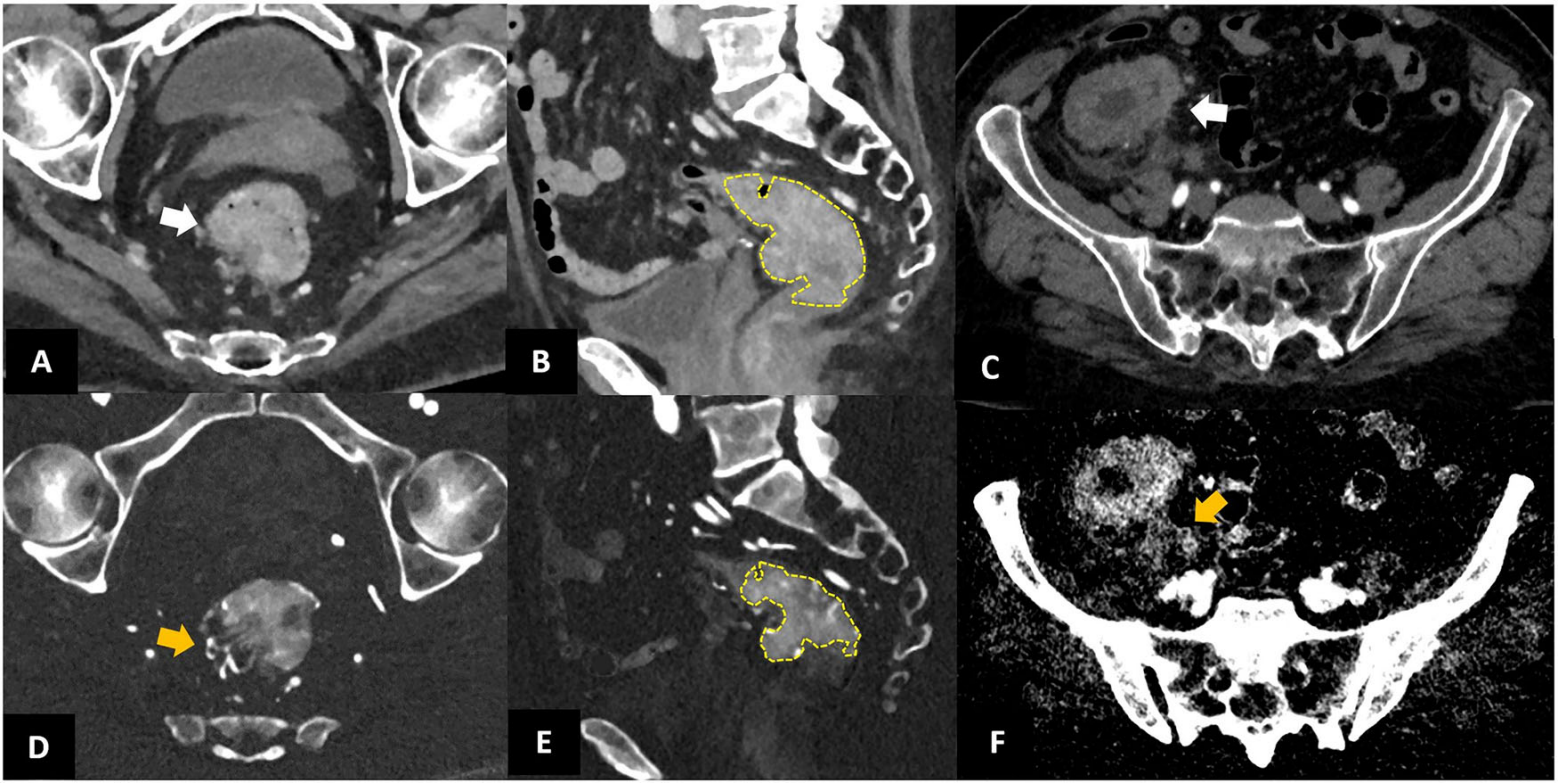
PCCT in colorectal cancer. Axial (**A**) and sagittal (**B**) post-contrast scan and the respective iodine maps (**D**, **E**) of a 93-year-old submitted to CT for weight loss. PCCT revealed a hypervascular tissue component at the sigmoid-rectal junction. Iodine maps (**D**, **E**) clearly identify the implantation base and the pedicle (orange arrow in **D**) with a more accurate evaluation of its intraluminal and craniocaudal extension (dashed yellow area in **E**) in comparison to the conventional scan (white arrows in **A** and dashed yellow area in **B**), enabling better discrimination of vascularized tissue from fecal content. Axial angiographic PCCT scan (**C**) and its iodine map (**F**) of an 86-year-old woman showing severe concentric thickening of the cecal walls, suggestive of neoplasia (white arrow in **C**), with a maximum thickness of approximately 2 cm and a craniocaudal diameter of about 8.5 cm, involving the ileocecal valve, and associated nodal metastasis. The iodine map improved identification of spiculation into the perivisceral adipose tissue, consistent with disease infiltration, and revealed iodine uptake of the edematous imbibition of the perivisceral adipose tissue surrounding the cecum, suggestive of a lymphangitic process (orange arrow in **F**)

However, the advent of PCCT has introduced several advantages in the evaluation of colorectal cancer, thanks to its improved soft tissue contrast and native spectral information [67]. In fact, generation of iodine maps improves the visualization of viable pathological tissue, with clear discrimination from fecal content, better definition of neoplasia extent, wall infiltration and involvement of peritumoral adipose tissue or vascular invasion (Fig. 7). Additionally, iodine maps allow for direct quantification of iodine contrast material [68], which has been shown to correlate with neoplastic thickness [69], and tumor lymphovascular invasion [70]. This makes it a potential biomarker of tumor shrinkage during treatment and a predictor of treatment response.

### Kidney and urinary tract

Kidney cancer is the 14th most common cancer worldwide, with renal cell carcinoma being the most prevalent subtype [71]. However, most incidental kidney findings are benign and small, and renal cysts are the most common. Discriminating between benign and malignant lesions can be challenging in many cases, with a reported 20% of surgically resected renal nodules proven to be benign [72]. While MRI offers superior soft tissue contrast and tissue characterization, without radiation exposure, CT is usually the first-line modality for the diagnosis and staging of renal masses [73].

The standard method to assess renal mass enhancement involves measuring the increase in attenuation between the unenhanced and contrast-enhanced phases. Generally, homogeneous renal masses of −9 to 20 HU on contrast-enhanced CT or > 70 HU on non-contrast CT can be classified as benign cysts [74]. However, this method is not always reliable, particularly when distinguishing between minimally enhanced renal cell carcinomas without visually solid components and hemorrhagic/proteinaceous cysts.

**Fig. 8:**
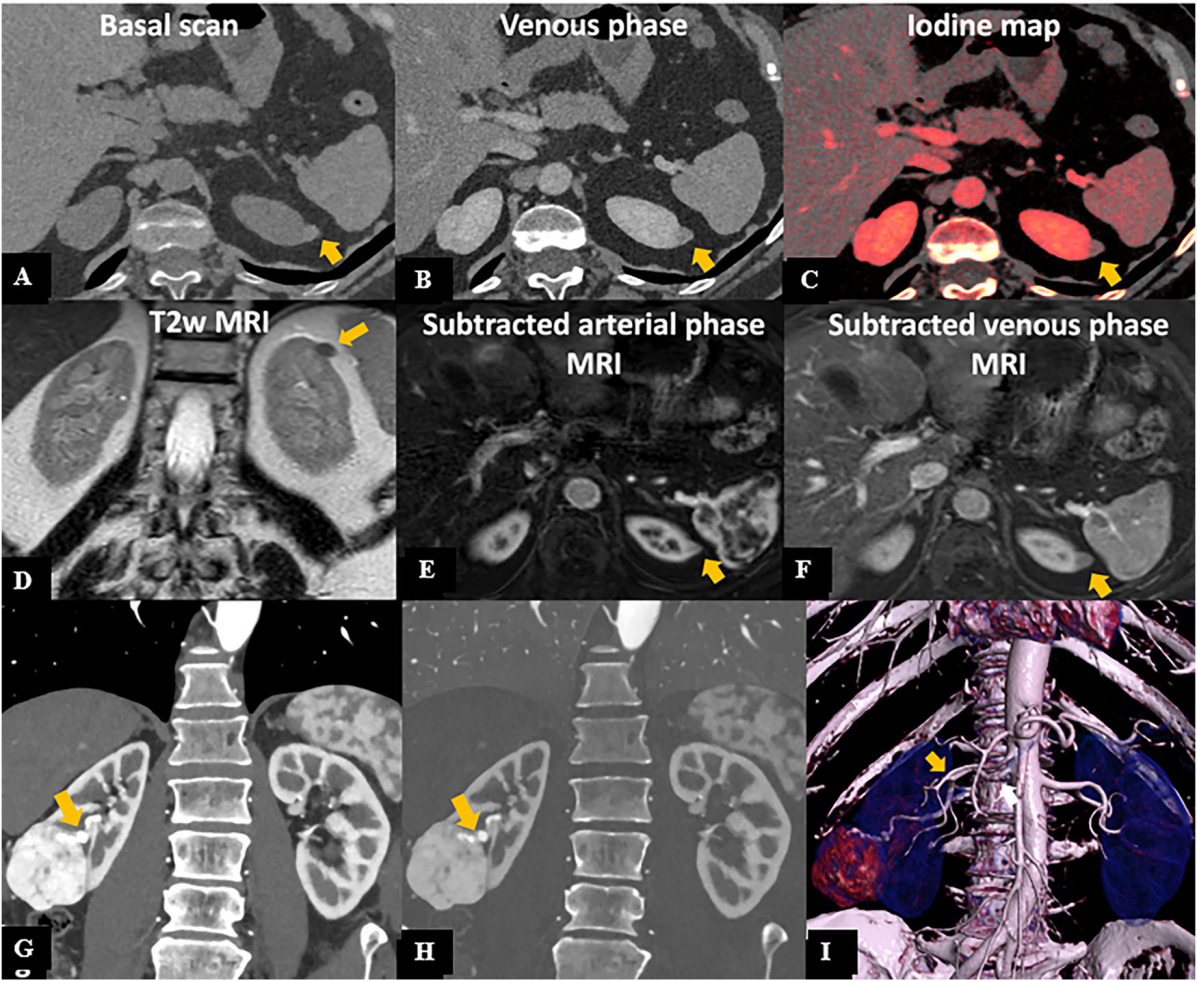
PCCT in renal cancer. A 57-year-old woman undergoing PCCT. Axial pre-contrast (**A**) and venous phase (**B**) scans show a small exophytic nodule of the superior pole of the left kidney (11 mm), slightly hyperdense in both phases, with mild iodine uptake at the iodine map (**C**), suggestive of a small papillary neoplasia rather than a hemorrhagic cyst. MRI confirmed the presence of a small left kidney nodule, hypointense in the T2w image (**D**), characterized by mild enhancement in the arterial phase (**E**), with minimal washout in the venous phase (**F**), thus confirming PCCT suspicion. PCCT in kidney tumor surgery planning: coronal angiographic CT scan showing an exophytic heterogeneous lesion in the mid-lower right kidney (**G**), 5 cm in diameter, with a well-defined hypertrophic vascular pole, further highlighted on the iodine map (**H**). 3D reconstruction shows a double right renal artery; the lesion is supplied by the more caudal branch (white arrow in **I**)

Iodine maps on PCCT can improve accuracy in differentiating enhancing from non-enhancing lesions, providing a better characterization of masses with equivocal enhancement as demonstrated in Fig. 8, in agreement with a previous study on EID-CT [75]. However, a unique threshold of iodine concentration has not been identified in DECT. This issue can be overcome with PCCT: a pilot study by Toth et al provided preliminary data on the upper limit of iodine concentration of non-enhancing renal lesions, thus paving the way for future spectral data application [76].

Subsequently, a pilot study by Becker et al [77] investigated the feasibility of PCCT in discriminating vascular from non-vascular hyperdense renal lesions without solid components through iodine and water material decomposition. They observed that an iodine concentration threshold of 20.3 HU enabled the discrimination of renal masses from proteinaceous/hemorrhagic cysts with > 87.5% sensitivity and specificity.

Another advantage of PCCT over DECT is the possibility to generate VMIs at any energy level, tailored to specific diagnostic needs. Low-keV VMIs are particularly useful in renal imaging, as they enhance renal parenchyma, increase contrast-to-noise ratio (CNR), and improve the detection of small enhancing structures such as septa or solid components. However, low-keV VMIs can also lead to pseudoenhancement of renal lesions, increasing the risk of misclassification. Unlike EID-CT, PCCT can correct beam hardening effects using spectral filtering, reducing pseudoenhancement and improving diagnostic accuracy. Shade et al [78] suggested that VMI at 70 keV provides an optimal balance between contrast enhancement of abdominal structures and accurate renal lesion assessment.

Finally, a recent prospective study comparing PCCT and MRI in patients with renal cell carcinoma showed that both modalities were comparable in identifying and characterizing several renal cancer types. Notably, MRI did not outperform PCCT in lesion classification [79].

Thus, the combination of iodine quantification and increased spatial resolution in PCCT may improve confidence and accuracy in characterizing small, otherwise indeterminate lesions compared to conventional CT and potentially even MRI.

Bladder cancer is the 10th most common cancer worldwide [80]. Accurate assessment of tumor local invasion is critical for treatment planning. It is necessary to discriminate between non-muscle-invasive bladder cancer and muscle-invasive bladder cancer. Currently, pre-operative cystoscopy biopsy is the standard of care; however, it is limited by the invasive nature of the procedure, its associated risks, and the potential for sampling error.

**Fig. 9:**
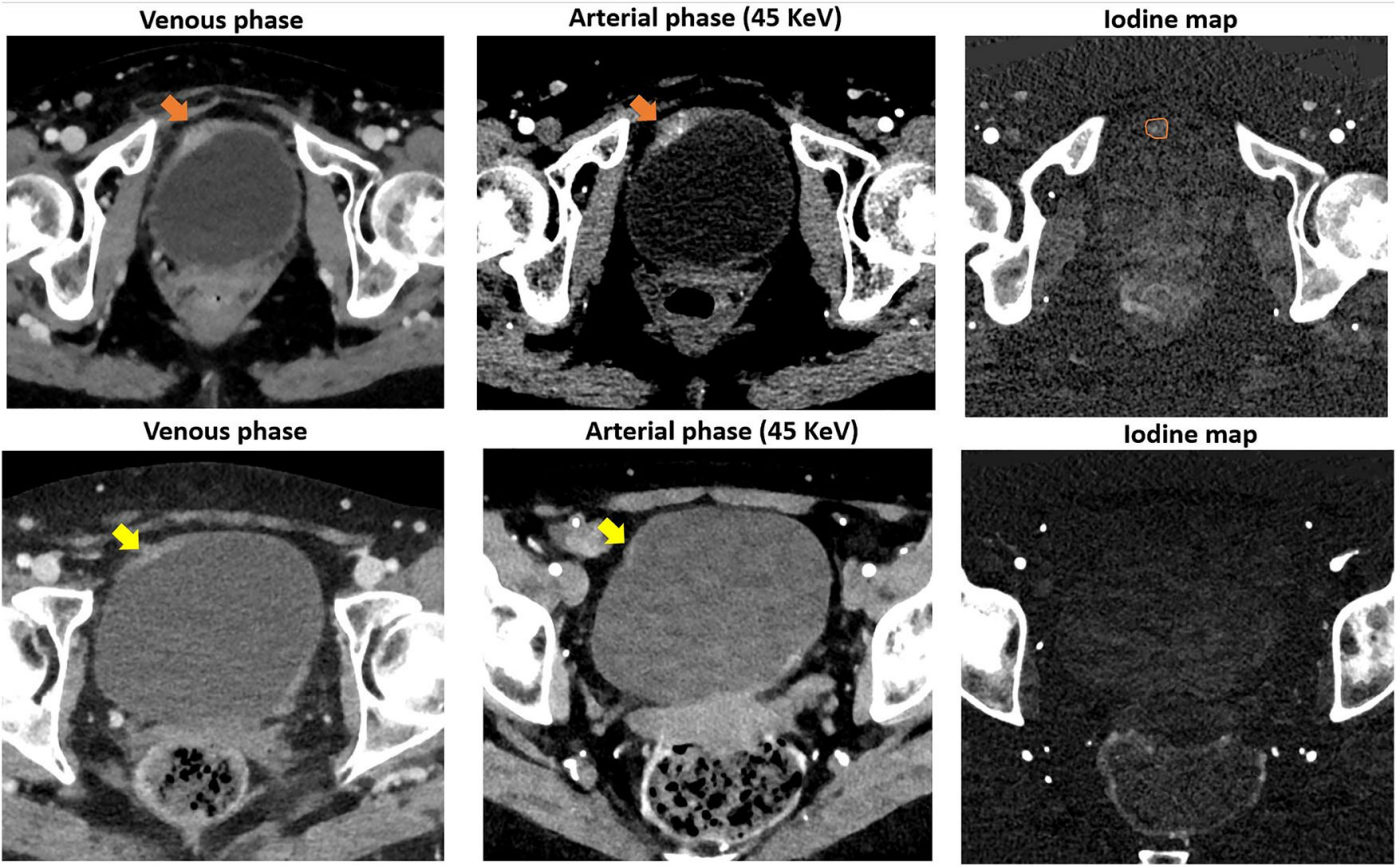
PCCT in bladder cancer. Two cases of patients undergoing PCCT after transurethral resection of bladder tumor. Top row: PCCT shows asymmetrical thickness in the anterolateral bladder wall with focal enhancement suspicious for relapse (orange arrows). The iodine map clearly identifies the area of pathological iodine uptake, which appears smaller in size compared to the arterial and venous phase (orange ROI). Bottom row: PCCT shows a focal thickening of the bladder anterolateral right wall (yellow arrows), with a thin superficial rim of increased enhancement, but without significant iodine uptake on iodine maps, suggestive of resection sequelae

Conventional EID-CT has limitations in detecting small tumors and in differentiating between T1 and T3 stages, as well as in distinguishing neoplasia relapse or resection sequelae. Preliminary data on PCCT suggest improved characterization of bladder wall involvement and potential aid in the assessment of muscle invasion (Fig. 9).

### Lymph nodes and peritoneum

Lymph node metastases (LM) detection is a crucial point in diagnostic, therapeutic planning and prognosis assessment of oncologic patients. To date, conventional CT diagnosis is based mainly on morphological features, especially including a short axis of more than 10 mm and the short-to-long axis ratio. However, this method is limited by its inability to detect micrometastases and the difficulty in distinguishing between inflammatory and malignant lymph nodes.

**Fig. 10:**
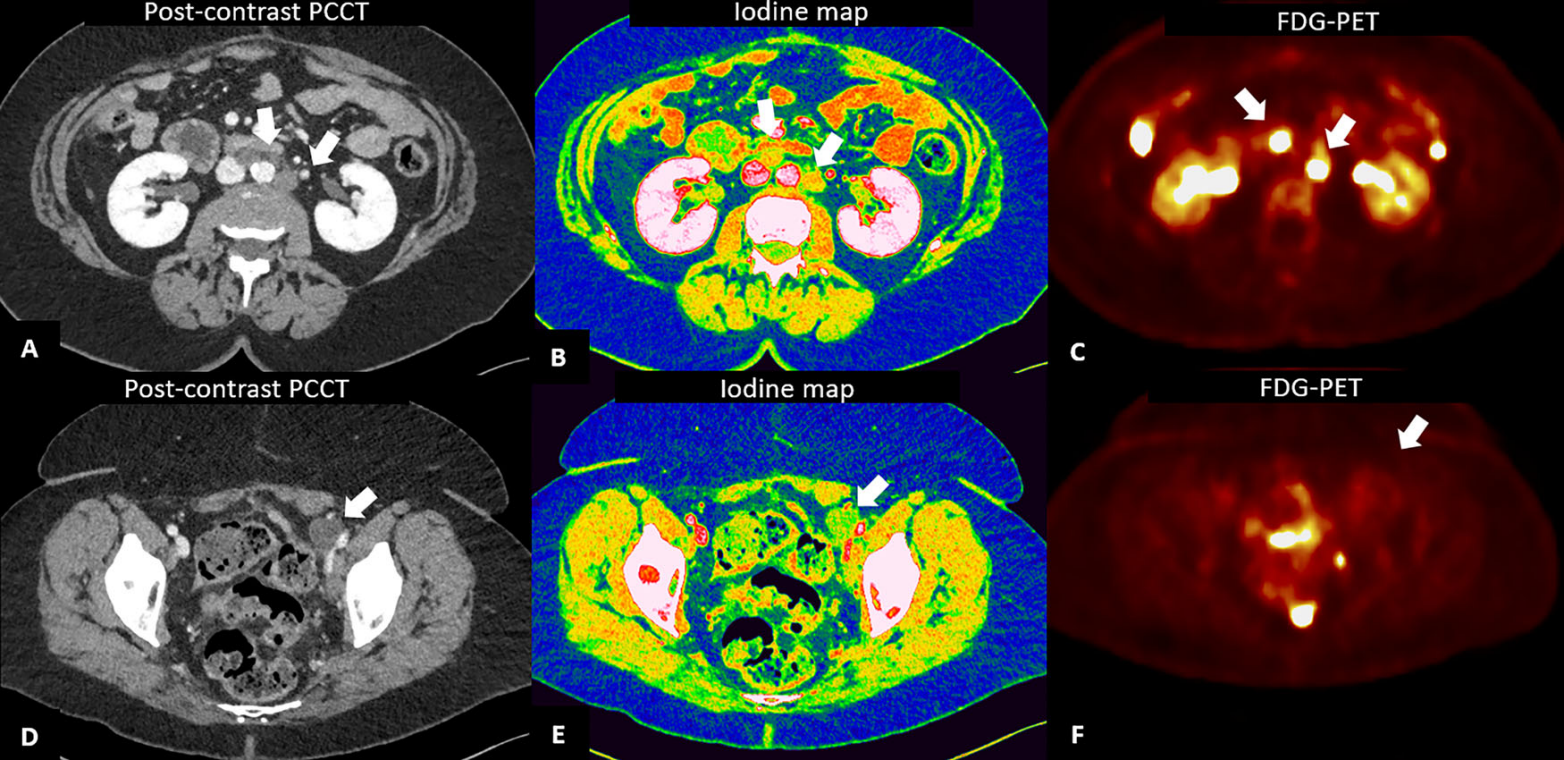
Lymph node metastases detection with PCCT. A 65-year-old woman with a history of oophorectomy for ovarian cancer 3 years prior, followed by immunotherapy for peritoneal carcinosis 2 years later, undergoing CT due to rising tumor markers and suspicion of cancer relapse. Angiographic axial scan (**A**–**D**) shows multiple rounded nodes along the abdominal aorta and iliac arteries, with a maximum short axis of 15 mm. The periaortic nodes showed iodine uptake on iodine map (**B**), suggestive of pathological involvement. In contrast, the left iliac node (arrow in **D**) shows no iodine uptake on the iodine map (**E**), primarily suggestive of lymphocele post-lymphoadenectomy. These results were confirmed at FDG-PET, which showed FDG uptake in the para-aortic nodes (**C**), and no uptake of the para-iliac node (**F**)

Spectral imaging represents a significant advancement in oncology, offering improved characterization of nodal metastases by providing iodine quantification, spectral attenuation curves, and VMIs, which can improve the differential diagnosis between metastatic and benign nodes (Fig. 10). Previous experience with DECT showed that iodine concentration and the slope of the spectral attenuation curve performed better than short-axis nodal diameter [81]. Similarly, Zhao et al [6, 82] found that DECT arterial-phase parameters might help in identifying occult nodal metastasis in patients with papillary thyroid carcinoma, while Zhou et al [83] demonstrated that the ratio of lymph node/tumor on 70-keV VMI aids in differentiating lymph nodes with and without metastasis more accurately than size measurement in patients with gastric cancer.

Although data on PCCT are still limited, Yalon et al demonstrated in a small cohort of patients with breast cancer that metastatic lymphadenopathy exhibited early avid enhancement similar to that of the primary tumor, which was better visualized with PCCT [35].

**Fig. 11:**
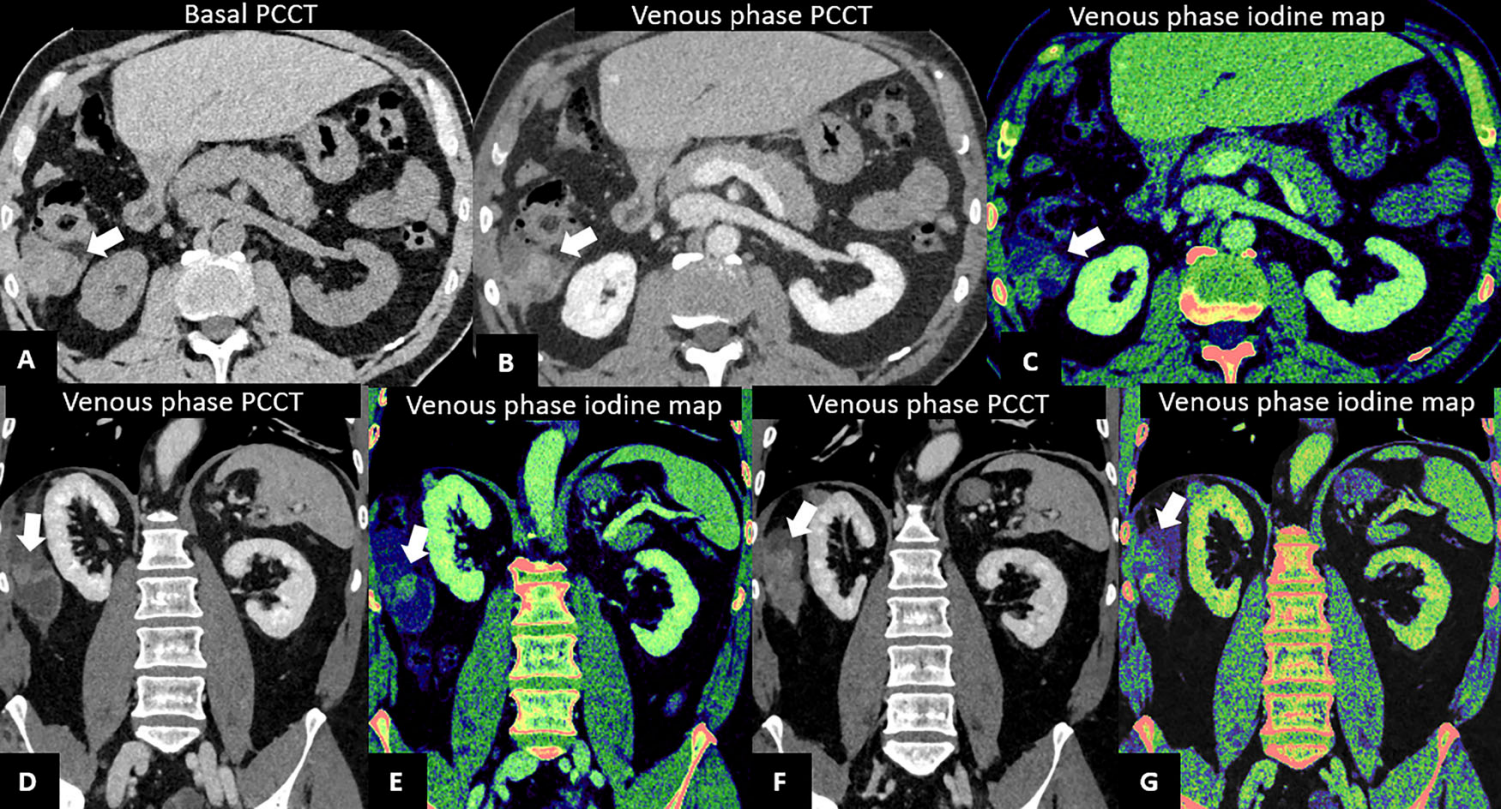
Peritoneum masses on PCCT. **A** 59-year-old man with a history of jejunal adenocarcinoma and liver metastasis, suspected of having a hematoma anterior to the right colon at the basal scan on EID-CT. Pre-contrast (**A**), post-contrast venous phase (**B**) axial and coronal views (**D**) show a fluid collection located in the right paracolic gutter, with a 3 cm solid tissue (white arrows in **A**–**G**), hyperdense on both basal and post-contrast scan, with evidence of iodine uptake on iodine maps (**C**, **E**), resulting referable to disease relapse. PCCT performed after 3 months demonstrated a significant increase in size and iodine uptake of the pathological tissue in the venous phase (**F**), more clearly visible in the iodine map (**G**)

Similarly, the identification of peritoneal metastases is a crucial aspect of patient management. A previous study demonstrated that DECT VMI at 40 keV improves the conspicuity of metastatic peritoneal deposits and radiologists’ diagnostic confidence compared with conventional CT [84]. However, a major advantage of PCCT over DECT lies in its ability to combine spectral images with significantly improved CNR, higher iodine sensitivity and superior spatial resolution, allowing for improved visualization of subtle peritoneal lesions. Additionally, low-energy VMI images and iodine maps enhance image quality and assist in distinguishing between benign and malignant peritoneal thickenings [85] (Fig. 11).

### Bone

**Fig. 12:**
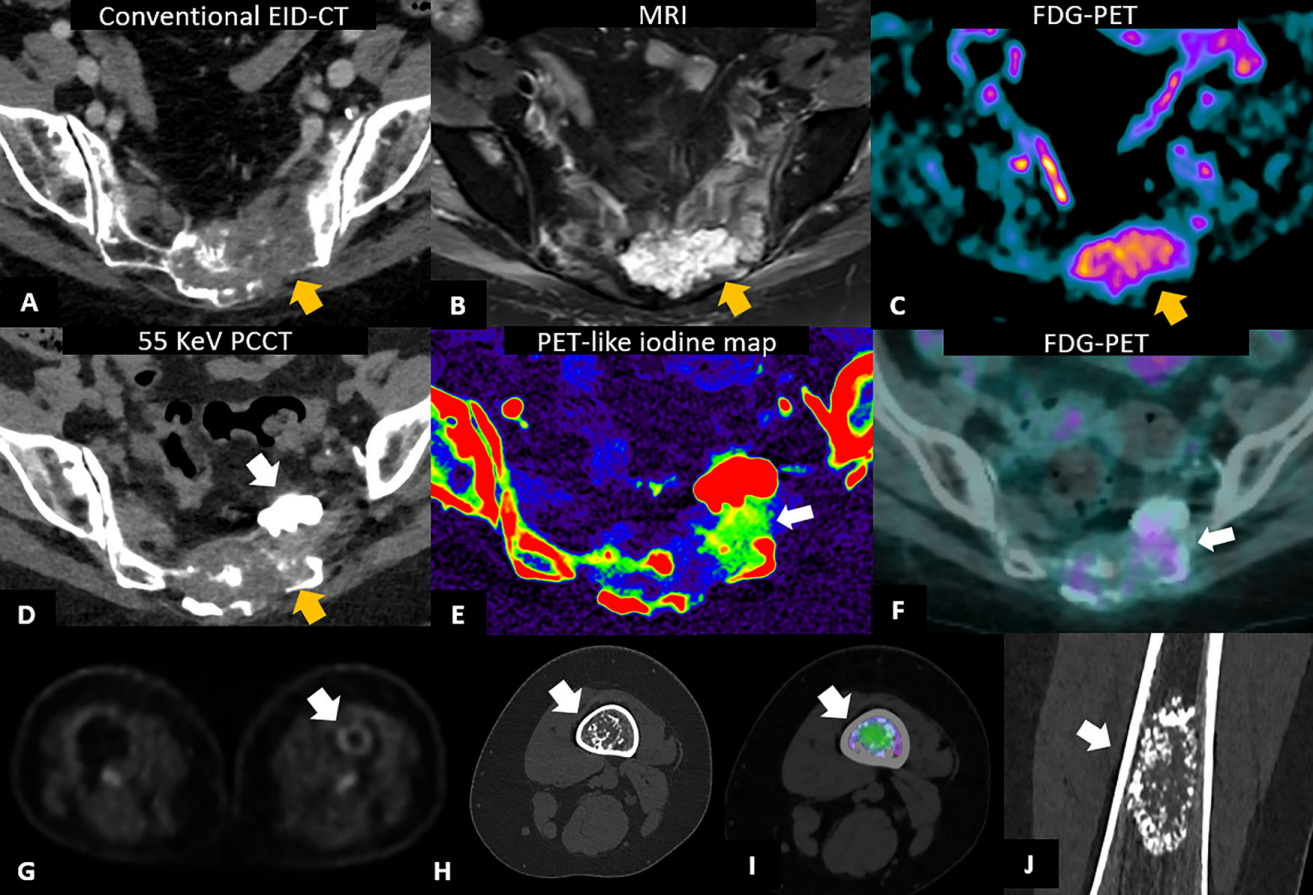
PCCT application in bone lesion. A 74-year-old woman with a history of breast cancer treated with surgery and RT, followed by systemic relapse and multiple bone lytic lesions (**A**) with cystic features at MRI (**B**) and iodine uptake on FDG-PET (**C**). Due to the risk of sacral fracture, the bone lesion underwent cryoblation and cementoplasty. PCCT performed after treatment showed ill-defined residual tissue (yellow arrow in **D**), without the possibility to discriminate between viable and non-viable metastatic components, along with the cementoplasty (white arrow in **D**). The iodine map (**E**) allowed identification of residual viable tissue (arrow in **E**), confirmed by FDG-PET (**F**). In the bottom row: a case of a 43-year-old woman with breast cancer undergoing FDG-PET (**G**) showing suspicious contrast uptake of the left femur. PCCT shows an oval lesion with honeycomb appearance (arrows in **H** and **J**) of 24 × 21 × 56 mm in size, at the distal diaphysis of the left femur, characterized by sclerotic edges and ipodense central component. Spectral evaluation shows intense central edema (green area in **I**) in the absence of perilesional edema. The multiparametric PCCT was suggestive of enchondroma

In musculoskeletal oncology as well, PCCT has shown several promising applications due to its high spatial resolution, with UHR images, combined with spectral information and material decomposition. This allows for improved bone marrow characterization in terms of edema and neoplastic involvement (Fig. 12) [86].

In particular, in a recent study by Baffour et al involving 27 patients with multiple myeloma, PCCT showed a significant increase in the detection of lytic lesions, intramedullary lesions, fatty metamorphosis, and pathologic fractures compared to EID-CT, even without the application of denoising by a convolutional neural network [87].

Additionally, Schwartz et al [88] found that in patients with multiple myeloma, PCCT and EID-CT were similar in terms of image quality, including cortical and trabecular delineation, image noise, diagnostic confidence, and osteolytic lesion detection, but PCCT achieved a 93% reduction in radiation dose.

Bone metastases are also frequent in oncology settings and are associated with significantly increased morbidity with mortality. Thus, early diagnosis is critical to guide patient treatment. PCCT may be particularly useful in identifying submillimeter bone lesions, which can be challenging with conventional CT. Moreover, the spectral capabilities of PCCT may aid in differentiating tumor progression from therapy-related changes (Fig. 12), particularly in cases of sclerotic evolution (i.e., pseudo-progression). In a recent study on bone metastases from breast cancer, PCCT was associated with reduced inter-reader variability in lesion size measurement and improved visualization of lesion margins and internal characteristics [89].

## Limitations and challenges

Most of the available studies are exploratory and have investigated the early diagnostic potential of PCCT in small samples, while data on long-term prognosis and validation in large cohorts of patients are still lacking. Unfortunately, to obtain robust data, broader adoption of the scanner is desirable, but high costs currently limit its availability. Networking among user sites is currently the most feasible option to hasten the increase of knowledge.

Moreover, PACS/infrastructure and archives may struggle to handle the volume of very large datasets resulting from ultra-high-resolution and spectral data, impacting review and storage efficiency. Therefore, investment in infrastructure is also required, resulting in financial challenges, especially for public healthcare systems.

Additionally, considering the recent introduction of this technology, research aimed at standardizing and optimizing image acquisition protocols, image reconstruction, and post-processing is needed. Similarly, the improvement in CNR calls for a revision of the contrast agent injection protocol, aimed at reducing contrast volume while maximizing attenuation. Minimizing contrast medium volume coupled with reduced radiation exposure would be a significant advantage in oncologic patients needing serial imaging.

Radiologists require training to maximize the potential of this novel technology in image acquisition and to correctly interpret multi-energy datasets, virtual monoenergetic images, and novel spectral reconstructions (e.g., iodine maps, virtual non-contrast). In the future, the development of systems with more than two energy bins, enabling richer spectral decomposition, would be beneficial in oncological settings, as well as the development of k-edge contrast agents for molecular imaging.

## Conclusions

PCCT represents a significant technological advancement in diagnostic imaging, offering enhanced spatial resolution, reduced radiation dose, and native spectral information. Preliminary data suggest a promising application of PCCT in the oncological field. PCCT has proven capable of providing superior lesion detection, improved tissue characterization, and greater diagnostic confidence compared to conventional EID-CT across a broad spectrum of oncologic applications, from liver, pancreas, kidney, and bladder to lymph nodes, peritoneum, and bone.

However, caution is needed in the interpretation of its true clinical benefits in the absence of large clinical validation. Despite the long road ahead, preliminary results are exciting, and PCCT seems to have what it takes to lead a revolution in precision imaging.

## Data Availability

Data included in the present manuscript were derived from published papers.

## Abbreviation

BCT: Breast computed tomography
CNR: Contrast-to-noise ratio
EID: Energy-integrating detector
HCC: Hepatocellular carcinoma
PCCT: Photon-counting detector computed tomography
PDAC: Pancreatic ductal adenocarcinoma
VMI: Virtual monoenergetic imaging
